# Epigenetic Regulation of Skeletal Tissue Integrity and Osteoporosis Development

**DOI:** 10.3390/ijms21144923

**Published:** 2020-07-12

**Authors:** Yu-Shan Chen, Wei-Shiung Lian, Chung-Wen Kuo, Huei-Jing Ke, Shao-Yu Wang, Pei-Chen Kuo, Holger Jahr, Feng-Sheng Wang

**Affiliations:** 1 Core Laboratory for Phenomics and Diagnostics, Kaohsiung Chang Gung Memorial Hospital, Kaohsiung 83301, Taiwan; ggyy58720240@gmail.com (Y.-S.C.); lianws@gmail.com (W.-S.L.); bulakuo@gmail.com (C.-W.K.); maggie2258tw@gmail.com (H.-J.K.); vip690221@gmail.com (S.-Y.W.); bank66882007@gmail.com (P.-C.K.); 2 Department of Medical Research, Kaohsiung Chang Gung Memorial Hospital, Kaohsiung 83301, Taiwan; 3 Department of Anatomy and Cell Biology, University Hospital RWTH Aachen, 52074 Aachen, Germany; hjahr@ukaachen.de; 4 Department of Orthopedic Surgery, Maastricht University Medical Center, 6229 HX Maastricht, The Netherlands; 5 Graduate Institute of Clinical Medical Science, Chang Gung University College of Medicine, Kaohsiung 83301, Taiwan

**Keywords:** epigenetic, osteoporosis, microRNA, histone modification

## Abstract

Bone turnover is sophisticatedly balanced by a dynamic coupling of bone formation and resorption at various rates. The orchestration of this continuous remodeling of the skeleton further affects other skeletal tissues through organ crosstalk. Chronic excessive bone resorption compromises bone mass and its porous microstructure as well as proper biomechanics. This accelerates the development of osteoporotic disorders, a leading cause of skeletal degeneration-associated disability and premature death. Bone-forming cells play important roles in maintaining bone deposit and osteoclastic resorption. A poor organelle machinery, such as mitochondrial dysfunction, endoplasmic reticulum stress, and defective autophagy, etc., dysregulates growth factor secretion, mineralization matrix production, or osteoclast-regulatory capacity in osteoblastic cells. A plethora of epigenetic pathways regulate bone formation, skeletal integrity, and the development of osteoporosis. MicroRNAs inhibit protein translation by binding the 3′-untranslated region of mRNAs or promote translation through post-transcriptional pathways. DNA methylation and post-translational modification of histones alter the chromatin structure, hindering histone enrichment in promoter regions. MicroRNA-processing enzymes and DNA as well as histone modification enzymes catalyze these modifying reactions. Gain and loss of these epigenetic modifiers in bone-forming cells affect their epigenetic landscapes, influencing bone homeostasis, microarchitectural integrity, and osteoporotic changes. This article conveys productive insights into biological roles of DNA methylation, microRNA, and histone modification and highlights their interactions during skeletal development and bone loss under physiological and pathological conditions.

## 1. Introduction

Osteoporosis is a common chronic skeletal disease with a plethora of deleterious conditions, such as extremely low bone mineral density, sparse microstructure, and poor biomechanical properties [1]. The degenerative bone disorder puts patients at a high risk of fracture, accounting for a major cause of lifelong disability or premature death [2]. Aging, menopause, hyperglycemia, glucocorticoid overmedication, etc., increase the prevalence of osteoporotic disorder [3]. Decreased osteoblastic activity and overdeveloped marrow adipose together with overactivated osteoclasts are prominent features, dysregulating bone formation and resorption activities in the osteoporotic skeleton microenvironment [4]. Bone-forming cells, such as osteoprogenitors, osteoblasts, and osteocytes, play an important role in maintaining skeletal tissue integrity. Understanding the emerging molecular mechanisms by which osteogenic cells lose mineral accretion capacity prompts us to have a deep insight into osteoporosis development.

Epigenetic pathways regulate transcription activity, without changing genomic DNA sequences [5], through a plethora of microRNA [6], enzymatic modification of 5-cytosine in DNA [7], post-translational modification of histones [8], and chromatin remodeling. DNA methyltransferases (DNMT) catalyze methylation of CpG islands of DNA sequences, decreasing genome stability to repress transcription activity [9]. MicroRNA interrupt mRNA targets to decrease protein translation or activate post-transcriptional signaling to upregulate protein expression [10,11]. Histone acetyltransferases (HATs) modify histone acetylation, which is indispensable in maintaining transcription, whereas histone deacetylases (HDACs) remove acetyl group of histones to favor the formation of heterochromatin that reduces promoter activity [12]. Histone methyltransferases catalyze methylation of lysine resides of histones to induce transcriptional repression [13]. Histone demethylases remove methyl group of lysine, reversing gene transcription [14]. In addition to enzymatic modification, many metabolites, such as butyrate, succinate, and propionate, are found to trigger histone butyrylation, succinylation, propionylation, and crotonylation [15]. The biological function of methylated DNA, microRNA, and histone modification for osteoblast behavior, bone tissue metabolism, and osteoporosis development warrants a collective review.

This article highlights how microRNA, histone modification, and DNA methylation affect osteoblast function and osteoporosis development and sheds light into how epigenetic modifiers for microRNA, histone acetylation, and methylation interact with microRNA, modulating osteoblastic activity and bone tissue integrity.

## 2. Organelle Machinery Regulating Osteoblastic Activity

Bone mass homeostasis is a dynamic process harmonized by bone forming cell-driven bone minerals that work together with osteoclast-controlled resorption. The former cell population produces growth factors and extracellular matrices indispensable in building up well-structured mineralized networks, as well as in contacting osteoclasts and secreting cytokines receptor activator nuclear factor-κ ligand (RANKL), osteoprotegrin (OPG), and chemokines, etc., to orchestrate osteoclastic activity [16]. Aging, estrogen deficiency, diabetes, and glucocorticoid excess in the skeletal tissue microenvironment are found to provoke intrinsic and extrinsic stresses on bone cells, interrupting intracellular organelle machinery and biochemical reactions that ultimately affect survival and metabolism [17].

### 2.1. Mitochondrial and Endoplasmic Reticulum Dysfunction Impair Osteoblast Behavior

Dysfunctional mitochondria are known to overproduce reactive oxygen radicals, destabilizing proteins or activating apoptosis programs in osteoblastic cells within the diabetes and age-mediated osteoporotic skeleton [18,19]. Advanced glycation end-products induce endoplasmic reticulum stress, accelerating osteoblast apoptosis in senile vertebrae [20]. The declined transportation capacity of the endoplasmic reticulum also decreases the transfer and distribution of mitochondria, suppressing intracellular homeostasis in senescent osteocytes [21]. Lysosomes are required to transport mineral-containing matrix vesicles out of osteoblastic cells [22], whereas the lack of lysosomal enzymes results in lysosome storage-mediated skeletal disorders [23]. Clearance of dysfunctional organelles is required to maintain biological activity of osteoblasts, osteoclasts, and skeletal health.

### 2.2. Defective Autophagy Induces Osteoblast Function

Autophagy is indispensable for getting rid of unwanted or dysfunctional organelles and macromolecules through autophagic puncta formation with p62, autophagy-related gene (Atg), and LC3 backbone and lysosomal degradation of the targeted organelles [24] during morphogenesis, remodeling, and senescence of various tissues [25,26]. Defective autophagy in osteoblasts hinders bone formation and remodeling [27]. Osteoblast-specific Atg5 knockout mice show severe osteoporosis phenotypes together with mineralized matrix underproduction and increased RANKL expression [28]. Mice lacking Atg7 in osteoblasts results in increased endoplasmic reticulum stress, along with sparse trabecular bone microstructure and poor osteogenic differentiation capacity of bone-marrow mesenchymal cells [29]. Upregulated autophagic reactions in osteocytes together with brittle skeletons are present in EphrinB2 knockout mice [30]. The 3-methyladenine inhibition of autophagy accelerates senescence programs of bone-marrow mesenchymal stem cells, decreasing growth and differentiation capacity. Reversing the autophagic reaction slows down bone loss in senile mice [31].

### 2.3. Autophagy of Mitochondria in Bone Cells during Osteoporosis

An interplay of autophagosome and mitochondria are required to sustain osteoblast function and bone mass. Mitophagy is an autophagic process of removing unwanted mitochondria to stabilize intracellular homeostasis. Loss of mitophagy worsens high glucose-mediated inhibition of osteogenic capacity and bone loss in diabetic mice. [32]. Mitochondrial chaperonin prevents autophagy regulator regulatory protein associated mammalian target of rapamycin (RPTOR) from glucocorticoid-induced conformation destabilization and ubiquitination, maintaining autophagic reaction and differentiation capacity in osteoblasts. Mice overexpressing mitochondrial chaperonin shows less bone mass loss and trabecular bone deterioration, as compared to wild-type mice [33]. The molecule also preserves mitochondrial function and survival of inflamed chondrocytes and also delays chondrocyte loss and cartilage erosion during osteoarthritis development [34]. While a plethora of genomic, biochemical, and biophysical factors control bone mass homeostasis and skeletal microstructure integrity, expanding evidence has revealed that epigenetic regulators, such as microRNA, DNA methylation and histone modification, also play important roles in bone health [35]. 

## 3. Genetic Variation, DNA Methylation, and Osteoporosis

Thanks to high throughput analytic technologies for genome-wide associated study, genetic variation of and DNA methylation are present in the development of human osteoporosis and osteoporotic fracture [16]. The former centers on genetic polymorphisms of bone regulators, like Wnt, estrogen receptor, and BMP. Epigenetic DNA methylation results in transcription repression, dysregulating osteoblastic activity and bone tissue metabolism. 

### 3.1. Single Nucleotide Polymorphism and Osteoporosis

Clinical evidence has revealed that over 500 gene loci are associated with bone tissue traits [36]. Genetic polymorphisms of bone regulators are relevant to low bone mass or osteoporosis. For example, single nucleotide polymorphisms in Wnt, β-catenin, and inhibitor sclerostin (SOST) are correlated with the development of human osteoporosis [37]. The vitamin D receptor polymorphisms change vitamin D pathways, which are associated with the development of osteoporosis [38]. A genome-wide associated study on over 1000 patients with menopausal women uncovered that locus rs10190845 at chromosome 2q13 is relevant to vertebral fractures, and that three loci at chromosome 1p31, 11q12, and 15q11 are associated with low bone mass [39]. 

### 3.2. DNA Methylation, Skeletal Homeostasis, and Osteoporosis

Increasing studies have shown that epigenetic modification controls bone cell survival, function, metabolism, and transformation during skeletal tissue development, remodeling, degeneration, and tumorigenesis. The methylation of the dinucleotide CpG site of the promoter is found to hinder transcription activity. DNA methylation enzyme DNMTs increase hypermethylation of DNA, whereas ten-eleven translocases (TETs) are found to induce DNA demethylation reactions by converting 5-methylcytosine to 5-hydroxymethylcytosine [9]. Osteoclast-specific DNMT3a knockout mice show high bone mass phenotypes and fewer osteoporotic conditions than wild-type mice. Likewise, osteoclast formation is downregulated in bone-marrow macrophage precursor cells deficient in DNMT3a [40]. Tet1 and Tet2 deletion result in defective osteogenesis of bone-marrow mesenchymal stem cells and severe osteoporosis [41]. Clinical evidence shows that the methylation state of CpG sites of zinc finger protein 267 (ZNF267), actin binding LIM protein family member 2 (ABLIM2), Ras homolog family member J (RHOJ), and cyclin dependent kinase like 5 (CDKL5) genes is correlated with postmenopausal osteoporosis [42]. Hypermethylation of CpG islands of bone-inhibitory factors, like SOST, dickkopf-1 (DKK1), and Wnt inhibitory factor 1 (WIF1) are present in postmenopausal women with osteoporosis [43]. Methylation of SOST gene together with increased SOST levels in serum and bone tissue are strongly correlated with osteoporotic fracture in postmenopausal patients [44]. Thanks to multi-omic analytical approaches, the development and progression of chondrosarcoma [45], osteosarcoma [46], and osteonecrosis of the femoral head [47] seem to be related to a wide hypermethylation of specific regions of the genome, with consequent loss of transcription of entire loci, both of miRNAs of different genes, as well as the activation of genes with high mitotic potential.

## 4. Effects of microRNA Actions on Osteoblast Function and Bone Integrity

MicroRNA (miR) are short non-coding, single strand RNA molecules with a molecular size around 18–25 base pairs. Precursor forms of microRNA are cut by endoribonucleases Drosha and Dicer into mature microRNA, which bind with Argonaute (AGO) proteins to form RNA-induced silencing complex [48]. MicroRNA are encapsulated by exosomes with double plasma membrane ultrastructures to protect them from degradation within the intercellular environment. The extracellular vesicles are a cargo of small molecules, like proteins, lipids, and noncoding RNA, which carry microRNA circulation around peripheral blood or facilitate the entry of microRNA into cells [49]. MicroRNA are found to inhibit mRNA expression and protein translation by binding the 3′-untranslated region of mRNA targets, regulating a plethora of biological activities in physiological and pathological contexts [50]. Evidence related to microRNA function in osteoblasts, osteoclasts, bone tissue, and human osteoporosis is still increasing. 

### 4.1. Biological Effects of MicroRNA Endoribonucleases on Skeletal Tissue

The biological function of microRNA-processing enzymes, like Dicer and Drosha, to bone tissue appears to depend on the differentiation potential of bone-forming cells or the genetic background of mice. For example, deletion of Dicer driven by osterix in osteoprogenitor cells in mice results in a high fetal mortality, whereas osteoblast-specific Dicer knockout mice show high bone mass [51]. The conditional deletion of Dicer in pre-osteoblasts in adult mice display decreased thickness and mechanical strength of cortical bone compared with wild-type mice [51]. While knockout of Dicer affects skeletal phenotypes, very little is revealed whether microRNA expression in bone tissue is changed in these animals. Osteoprogenitor cell-specific Drosha knockout mice driven by collagen I-Cre recombinase (Col1-Cre) show decreased miR-22 expression in the skeleton together with upregulated trabecular volume (BV/TV), trabecular thickness (Tb.Th), and trabecular number (Tb.N). Calcein-labeled mineral acquisition reaction in bone tissue is also increased in these knockout mice [52]. A study of Liu et al.’s group reveals that osteoblast-specific Dicer knockout mice driven by Runx2-Cre develop delayed bone development along with decreased microRNA expression, like let-7a, miR-27a, miR-101b, and miR-143. Alkaline phosphatase activity and mineralized matrix production of calvarial osteoblasts are inhibited in these animals [53]. In addition, cartilage-specific Drosha or DGCR8 deletion driven by Col2-Cre results in decreased miR-140, let-7b, let-7g, and miR-17 expression together with skull deformity and retarded longitudinal bone development. Conditional knockout of Drosha in articular chondrocytes driven by proteoglycan 4-Cre increases chondrocyte apoptosis and accelerates osteoarthritis development at the late stage [54].

### 4.2. MicroRNAs Affect Bone Tissue Integrity and Osteoporosis Development

Likewise, the biological responses of skeletal tissue to microRNA signaling depend on targeted mRNA or cell types in the bone microenvironment. Bone mineral density or trabecular morphometric characteristics of mice lacking miR-146a are similar to wild-type mice. Of interest, ovariectomized miR-146a knockout mice show less bone loss together with decreased RANKL and M-CSF expression and osteoclastic activity, as compared to ovariectomized wild-type mice [55]. CRISPR-Cas9 deletion of mmu-miR-185 promotes osteogenic differentiation of primary bone-marrow mesenchymal cells. Female miR-185 knockout mice with estrogen deficiency conditions display fewer osteoporosis signs than wild-type mice. miR-185 inhibits osteogenesis of osteogenic progenitor cells through the targeting of 3′-UTR of osteogenesis-promoting matrix biglycan [56]. Mice lacking miR-497–195 in endothelial cells display low bone mass along with decreased capillary vessel formation, whereas age-mediated bone loss and defective trabecular microarchitecture are delayed in mice overexpressing miR-497–195 in endothelial cells [57]. The microRNA is found to stabilize angiogenic activity by inhibiting angiogenic matrices, such as F-box and WD-40 domain protein (Fbxw7) and prolyl 4-hydroxylase possessing a transmembrane domain (P4HTM) [57]. Knockout of miR-188 reverses age-induced osteoporosis and fatty marrow. miR-188 overexpression in bone marrow mesenchymal cells worsens bone loss and marrow fat overproduction in senile mice. The microRNA directly targets HDAC9 and RPTOR-independent companion of MTOR complex 2 (RICTOR) to dysregulate osteogenesis and adipogenesis of bone-marrow stromal cells [58]. In addition, mice lacking miR-128 in osteoclasts develop high bone mass phenotypes and minor response to osteoclast overgrowth of bone-marrow macrophage cells and skeletal deterioration upon ovariectomy. miR-128 directly represses sirtuin 1 (SIRT1), the inflammatory cytokine interleukin-1β (IL-1β), and tumor necrosis factor-alpha (TNF-α) expression of osteoclastogenic progenitor cells [59]. Osteoclast-specific miR-214-3p knock-in results in increased serum exosomal miR-214-3p expression along with sparse trabecular bone structure. Inhibition of miR-214-3p in osteoclasts delays osteoporosis development in senile ovariectomized mice [60]. 

### 4.3. Serum MicroRNAs in Human Osteoporosis

The nature of exosomal microRNA secretion into peripheral blood rationalizes the feasibility to quantify serum microRNA levels and examine whether circulating microRNA is correlated with human osteoporosis. A cohort study revealed that serum miR-338 were increased in 15 postmenopausal women with osteoporosis, as compared to 15 healthy females. Receiver operating characteristic (ROC) curve analyses showed that the circulating microRNA was correlated with postmenopausal osteoporosis [61]. Analyses of the microRNA array showed that serum hsa-miR-122-5p and hsa-miR-4516 were decreased and correlated with osteoporosis in 139 patients [62]. A cross-section study showed that seven serum microRNA, namely miR-375, miR-532-3p, miR-19b-3p, miR-152-3p, miR-23a-3p, miR-335-5p, and miR-21-5p, were increased in 45 menopausal women with osteoporotic vertebral fracture. These serum microRNA were also correlated with serum bone markers [63]. A case-control study on 434 women uncovered that serum miR-26a-5p, miR-34a-5p, or miR-223-5p had nothing to do with osteoporotic fracture or abdominal aortic calcification [64]. Eight serum microRNA, including miR-18a-3p, miR-223-3p, miR-22-3p, miR-31-5p, miR-34a-5p, miR-143-5p, miR-423-5p, and miR-423-3p, were significantly changed in patients with osteoporosis inherited heterozygous WNT1 mutation [65]. Serum miR-33-3p and miR-133a, which putatively target RUNX2 and DKK-1, were changed in postmenopausal women with low bone mass upon anti-osteoporotic medication with teriparatide or denosumab [66]. While serum microRNA is relevant to osteoporosis development in postmenopausal women, rendering it as diagnostic markers or biosignatures requires more investigations with large sample sizes. The possibility cannot be ruled out that serum microRNA may not be extrapolated to the complex nature of the menopausal osteoporotic bone microenvironment, as the microRNA of interest may be secreted from other tissues. 

### 4.4. MicroRNAs in Bone Are Correlated with Human Osteoporosis

Expanding studies have revealed straightforward evidence of microRNA in human osteoporotic bone specimens. For example, an international multicenter cohort study with genome-wide analyses revealed that rs11614913T in the MIR196A2 gene in bone tissue was correlated with the risk of hip fracture. RNA sequencing analyses of MIR196A2 allele-transfected HEK293T cells uncovered that miR-196-5p targets 14 mRNA that are relevant to embryonic skeletal morphogenesis [67]. High throughput RNA-sequencing bone marrow from 33 postmenopausal women with osteoporosis and 22 menopausal women showed that miR-518b, miR-582-3p, miR-148a-3p, and miRNA-223-3p were increased in the osteoporosis group [68]. miR-213-3p expression in osteoclasts in bone marrow was upregulated in osteoporotic fracture bone [60]. Increased miR-128 expression in bone specimens was correlated with postmenopausal women with osteoporotic fracture [59]. miR-484, miR-328-3p, miR-27a-5p, miR-28-3p, and miR-409-3p expression in osteoporotic femoral bone specimens were correlated with bone mineral density of femur in 18 postmenopausal women [69]. Taken together, in studies on human osteoporosis, the extrapolation of serum microRNA levels to mirror microRNA expression in osteoporotic bone has not yet stood on common ground. 

### 4.5. The Biological Function of the miR-29 Family in Tissue Homeostasis

Of microRNA, human miR-29a and miR-29b are transcribed from sequences of chromosome 7 (7q32.3). miR-29c is reported to be from chromosome 1 (1q32.2) [70]. In rodents, the miR-29a family members are chromosomes 4q22, 13q27, 6qA3.3, and 1qH6. Bioinformatics searches from miRNet (www.mirnet.ca/miRNet/home.xhtml) reveal that a plethora of genes related to epigenetic modifiers and transcription factors, like HDAC4, DNMT3A, DNMT3B, RUNX2, PPARγ, and C/EBPα, are activated or repressed by human and murine miR-29a (Table 1). The bone-regulatory actions of the miR-29 family warrants review.

### 4.6. Roles of the miR-29a Family in Osteoporosis Development

Whole genome microarray analysis of our group revealed that a plethora of microRNA expression were changed in the glucocorticoid-mediated osteoporotic skeleton. miR-29a is decreased the most upon glucocorticoid treatment. Loss of miR-29a function accelerates bone mineralization loss and osteoclastic erosion [71]. We report that mice overexpressing of miR-29a display less glucocorticoid-induced bone mass loss, trabecular deterioration, and marrow adiposis [72]. Our group also revealed that osteoblast-specific miR-29a transgenic mice driven by osteocalcin promoter developed increased osteoblast growth and mineral accumulation in postnatal skeleton. The phenotypes of high bone mass and trabecular architecture prevent these adult animals from ovariectomy-induced osteoporosis and defective bone mechanics [73]. miR-29a inhibits osteoclast formation and resorption capacity through direct and indirect regulation of cytokines and immune regulators, like RANKL, tumor necrosis factor soluble factor 13b, and C-X-C motif chemokine ligand 12 (CXCL12). [73]. The miR-29 family are also found to modulate cartilage integrity and arthritic disease [74]. miR-29a and miR-29c regulate apoptosis of plasmacytoid dendritic cells from patients with Sjögren’s syndrome [75], as well as affect B cell behavior in collagen-induced arthritis [76]. 

## 5. Histone Modifications Control Skeletal Development

Histone stability is important to maintain chromatin structure in a lightly packed state, which sustains active transcription. Chemical modification of histones results in densely impacted chromatin that hinders polymerases for transcription. Histone acetyltransferases (HAT) and histone deacetylases (HDAC) modify the acetylation state of histones that are essential to active transcription [11,12]. Histone methyltransferases and demethylases regulate histone methylation that induces repressive transcription [13,14]. Increasing evidence has uncovered the function of these histone modifiers to bone mass homeostasis and structure integrity. 

Accumulating reports have revealed that metabolites activate post-translational butyrylation, succinylation, propionylation, and crotonylation at histone molecules [15]. Histone deacetylase Sirtulin (Sirt)-7 deletion results in lysine deacylation at osterix, inhibiting osteogenesis of bone-marrow mesenchymal stem cells and bone mass in mice [77]; however, little is still known about whether metabolite-mediated histone modification may influence osteoblast function, osteoclast behavior, bone mass homeostasis, and osteoporosis development. 

### 5.1. Histone Acetylation Is Required for Bone Formation

Histone acetyltransferase regulation of histone acetylation is indispensable in osteogenic capacity and bone formation. For example, knockdown of p300/CBP-associated factor (PCAF) reduces acetylation of lysine (K) 9 at histone 3 (H3K9ac), inhibiting osteogenic differentiation of mesenchymal stem cells and ectopic bone formation as PCAF RNAi-transfected osteoprogenitor cells are grafted into nude mice [78]. General control nonderepressible 5 (GCN5) signaling is decreased in the estrogen deficiency-mediated osteoporotic skeleton. GCN5 interference impairs the osteogenic commitment of mesenchymal progenitor cells and ectopic skeletal development [79]. Jing et al. showed that GCN5 increases H3K9ac as well as promotes the acetyl histone binding promoters for Wnt proteins in osteogenic progenitor cells. Forced GCN5 expression through the lentivirus shuttle slows ovariectomy-mediated loss of bone mineral density, trabecular morphometry, dynamic bone formation histology, and osteogenic differentiation of bone-marrow mesenchymal stem cells [80]. 

### 5.2. Histone Deacetylases Regulate Skeletal Development 

The biological functions of HDAC to bone tissue or osteoporosis development likely depends on HDAC types or the developmental potential of the skeleton. In bone development, mice lacking HDAC3 in osteoprogenitor cells showed perinatal morality, defective limb development, and poor osteoblast differentiation [81]. HDAC4 knockout in osteoprogenitor cells driven by osterix-Cre or osteochondrogenic cells driven by Col2-Cre resulted in marrow adiposity in the bone microenvironment and high adipocyte formation of bone-marrow mesenchymal progenitor cells [82]. Loss of HDAC4 induces premature ossification during skeletal development, whereas gain of HDAC4 decreases chondrocyte hypertrophy and ossification [83]. HDAC4 mediates Ca^2+^ channel transmembrane and coiled-coil domains 1 (TMCO1) knockout-induced low bone mass and osteogenic differentiation [84]. Mice deficient in HDAC5 display decreased trabecular bone volume and bone mineralization together with increased SOST expression in osteocytes [85]. HDAC7 knockout mice driven by Col2-Cre display low trabecular bone volume (BV/TV) and trabecular number (Tb.N) together with unaffected trabecular thickness (Tb.Th) or cortical bone microstructure. [86]. Sirtuin-1 (Sirt1) modulates oxygen sensor prolyl hydroxylase 2 deficiency-mediated high bone formation capacity and bone mineral density [87]. Decreased Sirt1 expression together with adipocyte overgrowth at the cost of osteogenic differentiation are present in bone-marrow stromal cells from mice with chronic energy restriction. Gain of Sirt1 signaling improves osteogenesis in anorexia-mediated osteoporotic bone [88]. 

### 5.3. Histone Methylation Alters Bone Formation and Remodeling 

Post-translational methylation of lysine residues of histones changes chromatin structure into a heterochromatin that interrupts active transcription. A plethora of histone methyltransferases and demethylases catalyze the modification processes. Increasing evidence sheds new light onto the epigenetic actions of trimethyl H3K27 (H3K27me3) to tissue development and deterioration. Of the H3K27me3 modifiers, histone methyltransferases polycomb repression complex 2 (PRC2) subunits zeste homolog 2 (EZH2), embryonic ectoderm development (EED), and SUZ12 catalyze H3K27 trimethylation. Ubiquitously transcribed tetratricopeptide repeat X chromosome (UTX) and Jumonji domain containing 3 (Jmjd3) were found to erase the trimethyl group of the histone [13]. The impact of these epigenetic modifiers on bone tissue warrants review.

Mice deficient in Ezh2 in osteoprogenitor cells driven by Prrx-Cre display impaired bone development. Ezh2 loss in chondrocytes driven by Col2-Cre results in low bone mass in the adolescent rather than the adult stage; however, Ezh2 or H3K27me3-binding epigenomic marks in knockout mice are comparable to wild-type mice [89]. Mice lacking Ezh1 and Ezh2 driven by Col2-Cre develop smaller bone phenotypes with decreased chondrocyte proliferation. Comparative transcriptome reveals cyclin-dependent kinase inhibitor 2a/b (Cdkn2a/b) and insulin-like growth factor binding protein (Igfbp) signaling that mediate Ezh1 and Ezh2 control of skeletal development [90]. Osteoblast-specific Ezh2 knockout mice driven by Osx-Cre display skeletal phenotypes similar to wild-type mice, while trabecular bone volume, osteoblast surface, and osteogenesis of bone-marrow mesenchymal cells are decreased in young knockout mice [91]. Deletion of EED in mouse oocytes driven by Zp3-Cre results in skeletal overdevelopment with increased bone mineral density [92]. Histone methyltransferase SET-domain-containing 2 (SETD2) modifies H3K36 trimethylation and promotes the trimethyl histone binding promoters for lipopolysaccharide-binding protein (LBP), modulating adipogenic and osteogenic specification. SETD2 knockout drives mesenchymal stem cells into adipocytes at the expense of osteoblast differentiation. Setd2 knockout mice in osteoprogenitor cells show low trabecular volume and bone formation rate together with marrow fat overproduction [93]. 

### 5.4. Histone Demethylation Influences Osteogenesis and Adipogenesis in Bone Tissue

In addition, histone demethylase UTX is found to interact with EZH2, regulating osteogenesis and adipogenesis of human mesenchymal stem cells. EZH2 interference inhibits adipocyte formation, whereas UTX knockdown upregulates adipogenic differentiation [94]. Histone demethylases KDM4A and 4B remove trimethyl groups of H3K9 and H3K27, respectively. Knocking down KDM4A or 4B promotes adipocyte formation, whereas osteogenic specification is suppressed [95]. Kdm3a knockout mice show worsened periodontitis-induced alveolar bone destruction together with increased osteoclasts along eroded bone tissue [96]. GEO database of microarray analysis reveals that KDM5A expression is increased in bone marrow mesenchymal stem cells from human osteoporosis. Forced KDM5A expression reduces H3K4me3 together with increased osteogenic differentiation. KDM5A interference or inhibitor reverses estrogen deficiency-mediated loss of bone mass and mineral accretion [97]. UTX loss is present in glucocorticoid-mediated osteoporotic bone tissue. UTX knockdown attenuates glucocorticoid-mediated adipocyte formation, reversing osteogenesis of mesenchymal stem cells by increasing H3K27me3 binding Runx2 and osterix promoters. Likewise, inhibition of UTX worsens bone mass loss in glucocorticoid-treated skeletal tissue [98].

### 5.5. Interplay of miRNAs and DNA and Histone Modifications in Bone Tissue

**Figure 1:**
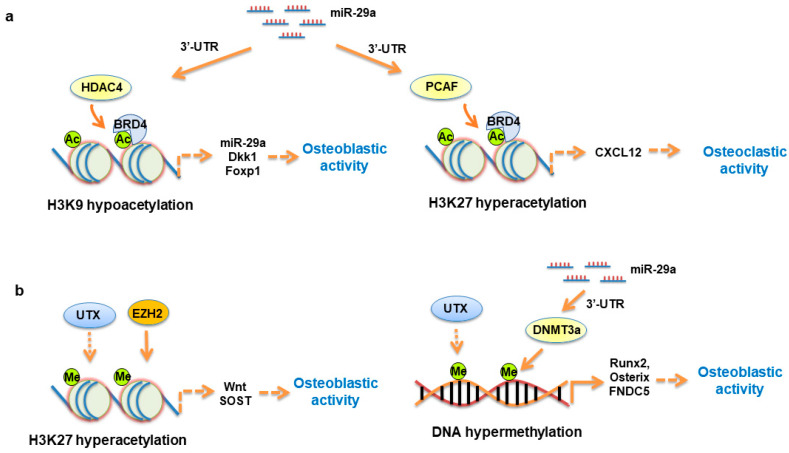
Schematic drawing of miR-29a, acetyl histones, and methyl histone regulation of osteoblast function and bone integrity. miR-29a targets HDAC4, increasing H3K9ac binding promoters for miR-29a and Dkk-1. H3K9ac interacts with BRD4 to affect Foxp1 transcription. miR-29a targets PCAF, decreasing H3K27ac enrichment in CXCL12 promoter (**a**). miR-29a interacts with UTX and EZH2, regulating H3K27me3 binding promoters for Wnt and SOST to modulate osteogenic differentiation. UTX modifies methylation of Runx2, osterix, and FNDC5 promoters to regulate osteogenic differentiation capacity (**b**).

The interaction of microRNA and histone modification is also present during osteogenic differentiation or osteoporosis development. The gain of miR-7-5p function promotes p300-mediated histone 3 hyperacetylation, increasing Runx2 transcription and osteogenic differentiation [99]. miR-23 targets homeobox protein (HOX) signaling along with reduced H3K18ac and H3K27ac binding promoters for bone alkaline phosphatase, osteocalcin, and Runx2, inhibiting mineralization capacity of osteoblastic cells [100]. miR-99a binds 3′-UTR of KDM6B, blocking osteogenic differentiation. Exogenous miR-99a mimic inhibits bone formation of cranial bone defects [101]. We showed that decreased miR-29a together with increased HDAC4 levels are present in mesenchymal stem cells from osteoarthritic subchondral bone. Forced miR-29a expression inhibits HDAC4 expression, counteracting mineralized matrix overproduction [102]. Gain of miR-29a function decreases PCAF-mediated H3K27ac binding CXCL12 promoter, reducing the cytokine secretion for osteoclast formation [73]. miR-29a targets HDAC4, increasing H3K9 acetylation to ward off glucocorticoid excess-induced loss of Wnt signaling components and osteoblastic activity. HDAC4 knockdown increases H3K9ac enrichment in miR-29a promoter, upregulating miR-29a expression in osteoblastic cells [103], as shown in Figure 1a. In addition, miR-29a interacts with UTX, which decreases H3K27m3 binding promoters for Wnt and Dkk-1 and downregulates CpG methylation at promoters for Runx2 and osterix during glucocorticoid-mediated osteogenesis loss and adipocyte formation (Figure 1b).

## 6. Therapeutic Potential of Epigenetic Modifiers as Treatment for Osteoporosis

The epigenetic actions to bone formation and remodeling reactions facilitate the development of epigenetic therapeutic potential for osteoporotic skeletons. A wide array of proof-of-concept studies have revealed the remedial effects of microRNA and epigenetic modifiers to slow osteoporosis development. Recombinant adeno-associated virus shuttled artificial microRNA targeting RANK ameliorates osteoclast formation and estrogen deficiency and age-induced osteoporosis in mice [104]. Intravenous injection of miR-338 inhibitor preserves bone formation capacity, improving bone mass and trabecular structure in ovariectomized mice [61]. Administration with miR-672-5p increases bone mineral accretion, repressing bone mass loss and sarcopenia in ovariectomized mice [105]. Inhibition of histone methyltransferase DOT1L decreases osteoclastic resorption, delaying osteoporosis development [106]. Treatment with EZH2 inhibitor drives bone-marrow mesenchymal cells away from adipocytes, reversing osteogenic differentiation capacity to downregulated osteoporosis [107]. DNA methylation inhibitor 5-aza-2′-deoxycytidine ameliorates disuse-induced osteopenic bone development [108]. While genetic and pharmacological modulation of microRNA, DNA methylation, and histone modification have protective effects to experimental osteoporosis, the perspective of these epigenetic modifiers for repressing human osteoporotic disorders remains elusive.

## 7. Conclusions

This review delivers collective insights into what roles DNA methylation, microRNA, and histone modification play in skeletal tissue development and homeostasis. Evidence from transgenic and knockout mouse models reveals that these epigenetic regulators appear to act as game changers or bystanders for bone formation and deterioration, depending on bone cell types and intrinsic and extrinsic stresses on bone microenvironments. While studies of proof-of-principle show that serum microRNA levels are likely correlated with human osteoporosis, multicenter cohort investigations with more powerful sample sizes warrant further confirmation for adopting serum microRNA as diagnostic markers for osteoporosis. Interaction of miR-29a, HDAC4, H3K9ac, and H3K27ac in osteoblasts are indispensable in sustaining mineralized matrix synthesis and osteoclast-regulatory chemokine production for protecting bone tissue from the development of glucocorticoid excess or estrogen deficiency-mediated osteoporosis. This article sheds light onto the physiological, pathological, and clinical significance of epigenetic pathway in skeletal health.

## Figures and Tables

**Table 1:** Putative transcription factor targets of human and murine miR-29a.

Transcription Factor Targets	hsa-mir-29a	mmu-mir-29a
Activation	AP-1, E2F1, FOXA2, HMGA1, NFE2L2, NR1H4, STAT1, STAT3, STAT5B,	Hnf4a, Nfe2l2
Repression	DNMTA, DNMT3B, GLI1, HDAC4, IL4, NFKB1, MYC, PDGF-B, TP53, TGFB1, SOX9	Hdac4, Pparg
Regulation	CEBPA, MITF	Ar, Cebpa, Cebpb, Ebf2, EP300, Erg, Esr, Fli1, Foxl2, Nr3c1, Onecut 1, Prdm16, Rela, Runx1, Stat1, Stat5, Tgifl, Trim33
Activation or repression	AR, CEBPB, FOXA1, FOXH1, GTF2I, NOTCH1, MYB, PGR, RUNX2, SOX2, SUMO, YY1	Brd4, Dmc1, Gata3, Gata2, Med1, Rxra, Spil

mRNA targets are predicted by bioinformatic searches of miRNet (www.mirnet.ca/miRNet/) with the permission.
